# The binary aluminum scandium clusters Al_*x*_Sc_*y*_ with *x* + *y* = 13: when is the icosahedron retained?

**DOI:** 10.1039/d1ra06994b

**Published:** 2021-12-16

**Authors:** Ngo Tuan Cuong, Nguyen Thi Mai, Nguyen Thanh Tung, Ngo Thi Lan, Long Van Duong, Minh Tho Nguyen, Nguyen Minh Tam

**Affiliations:** Faculty of Chemistry, Center for Computational Science, Hanoi National University of Education Hanoi Vietnam; Institute of Materials Science, Graduate University of Science and Technology, Vietnam Academy of Science and Technology 18 Hoang Quoc Hanoi Vietnam; Department of Physics and Technology, Thai Nguyen University of Science Thai Nguyen Vietnam; Institute for Computational Science and Technology (ICST) Quang Trung Software City Ho Chi Minh City Vietnam; Department of Chemistry, KU Leuven Celestijnenlaan 200F B-3001 Leuven Belgium; Computational Chemistry Research Group, Ton Duc Thang University Ho Chi Minh City Vietnam nguyenminhtam@tdtu.edu.vn; Faculty of Applied Sciences, Ton Duc Thang University Ho Chi Minh City Vietnam

## Abstract

Geometrical and electronic structures of the 13-atom clusters Al_*x*_Sc_*y*_ with *x* + *y* = 13, as well as their thermodynamic stabilities were investigated using DFT calculations. Both anionic and neutral isomers of Al_*x*_Sc_*y*_ were found to retain an icosahedral shape of both Al_13_ and Sc_13_ systems in which an Al atom occupies the endohedral central position of the icosahedral cage, irrespective of the number of Al atoms present. Such a phenomenon occurs to maximize the number of stronger Al–Al and Sc–Al bonds instead of the weaker Sc–Sc bonds. NBO analyses were applied to examine their electron configurations and rationalize the large number of open shells and thereby high multiplicities of the mixed clusters having more than three Sc atoms. The SOMOs are the molecular orbitals belonged to the irreducible representations of the symmetry point group of the clusters studied, rather than to the cluster electron shells. Evaluation of the average binding energies showed that the thermodynamic stability of Al_*x*_Sc_*y*_ clusters is insignificantly altered as the number *y* goes from 0 to 7 and then steadily decreases when *y* attains the 7–13 range. Increase of the Sc atom number also reduces the electron affinities of the binary Al_*x*_Sc_*y*_ clusters, and thus they gradually lose the superhalogen characteristics with respect to the pure Al_13_.

## Abstract 2

The icosahedral structure of the Al*_x_*Sc*_y_* clusters with *x* + *y* = 13.

## Graphical abstract

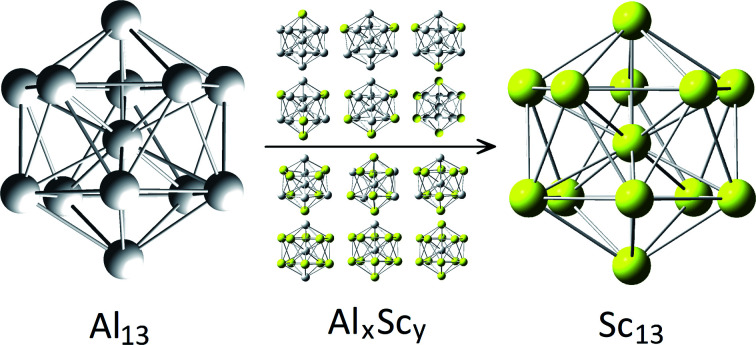

## 1. Introduction

During the last four decades, a very large number of both experimental and theoretical studies on atomic clusters were reported with the aim to understand their novel physical and chemical properties as well as to emphasize their abilities to be used for new promising technological applications.^1^ The atomic clusters possessing high symmetry geometries have often attracted more attention, in part due to the fact that they are expected to have an enhanced stability along with appealing features. In particular they can be considered as building blocks for assembled nanostructured materials.^1,2^ Especially, many investigations found that various clusters formed by 13 atoms, including both homonuclear and heteronuclear derivatives, exist in an icosahedral shape and have interesting physico-chemical properties that could lead to significant applications.^3^ For instance, to adjust the overall valence state and thereby the chemical behavior of the silicon doped aluminum Al_12_Si icosahedron, it is possible to substitute the Si atom by another dopant such as B, P and Ca, to form the superhalogen Al_12_B, super alkali metal Al_12_P, and superchalcogen Al_12_Ca, respectively.^4^ Of the main group element clusters, only the aluminum clusters at this size, Al_13_, in both neutral and anionic states, are icosahedra formed by 13 homonuclear atoms,^5–7^ that are one of the most well-known and inspiring example for superatomic clusters.^1^ The anion Al_13_^−^ exhibits 40 valence electrons in a closed shell structure and thus emerges as a *magic cluster* with an enhanced thermochemical stability. For its part, the neutral Al_13_, having one valence electron less than the anion Al_13_^−^, has a very high electron affinity exceeding those of halogen atoms and has been regarded as a superhalogen.^8,9^ Stimulated by the existence of the icosahedral Al_13_, many similar structures were found by doping of various hetero-atoms into aluminum hosts.^3^ A previous study using photoelectron spectroscopy combined with theoretical calculations pointed out that an icosahedral anion Al_12_Li^−^ can be composed by replacing a surface Al atom of the icosahedron Al_13_^−^ by a Li atom.^10^ For the doped aluminum Al_12_X^2−^, while the beryllium, X = Be, sits at the center of an aluminum icosahedral cage due to its small atomic radius, the remaining dopants with X being an alkaline earth metal having larger atomic radius, favor its attachment outside at the surface.^11–13^ Although the aluminum doped boron clusters B_12_Al favor a quasi-planar shape,^14^ it was also found that other dopants which belong to the same group IIIA as aluminum including B and Ga, can substitute the Al atom(s) at the center of icosahedral cage Al_13_.^15,16^ An investigation of the cationic clusters Al_12_X^+^, with X being a tetravalent atom including C, Si, Ge, Sn, and Pb, showed that except for the low symmetry structure of Al_12_C^+^, the remaining structures of Al_12_X^+^ prefer an icosahedral shape. The Si and Ge atoms are encapsulated at the central position of the icosahedra Al_12_Si^+^ and Al_12_Ge^+^, respectively, whereas for the Al_12_Sn^+^ and Al_12_Pb^+^, the dopant substitutes an aluminum atom on a vertex of the icosahedral framework.^17^ Moreover, a recent study on singly and doubly silicon doped aluminum clusters reported that both neutral and cationic states of Al_11_Si_2_ keep on favoring the icosahedral shape with one Si dopant embedded at the central position, whereas the remaining Si atom substitutes an external Al position of the icosahedron Al_13_.^18^ Similarly, another study of Al_12_X clusters at both neutral and cationic states, with dopant X being a pentavalent atom including P, As, Sb and Bi, also showed that the P atom prefers to be at the center of the Al_12_ icosahedron whereas the rest of the dopants favor occupancy of a vertex site due to their larger size.^19^

Previous studies on transition metal doped aluminum clusters also reported that an icosahedral structure can be composed by 12 aluminum atoms plus a transition metal dopant such as Co, Ni, Cu, and Zn. The atomic radius of the latter seems small enough to allow its position at the center of such a cage.^10,11,20–22^ A combined theoretical and experimental study on Al_*n*_V clusters^23^ showed that the anionic Al_12_V^−^ prefers a distorted icosahedral shape in which the vanadium atom occupies a convex capped site. Interestingly, Kumar and Kawazoe carried out a study using density functional theory (DFT) calculations on a doping of a Cu atom into Al_12_ and discovered a perfect icosahedral Cu@Al_12_ possessing a high spin state with a 3 *μ*_B_ magnetic moment.^22^ A subsequent theoretical study found that the Al_12_Cu could play as a stable building block to form ionic salts, as shown from stable dimers, trimers, and tetramers of the Al_12_CuM_3_ complex.^24^

On the other hand, it appears that an icosahedral structure based on the elements of the main group IVA can be also obtained upon doping of an impurity atom onto a 12 tetravalent atom system. A theoretical study indicated that the most stable isomers of the Ge_12_M^*x*^ clusters, with M = Li, Na, Be, Mg, B and Al with *x* going from −1 to +1 and each containing 50 valence electrons, prefer a high symmetry icosahedron.^25^ Particularly for the anion Ge_12_Li^−^, the lithium dopant was found at the central position of the icosahedral cage instead of on its surface as in the case of Al_12_Li^−^.^25^ Goicoechea and McGrady carried out a theoretical investigation of endohedrally transition metal doped silicon MSi_12_ and germanium MGe_12_ clusters, and found that while MSi_12_ have a hexagonal prism or a bicapped pentagonal prism shape, some MGe_12_ with M being Cr, Mn, Fe, Cu, Zn, Ag, and Cd, tend to favor an icosahedral form.^26^ Similarly, many icosahedra bearing an endohedrally doped metal such as tin MSn_12_ and lead MPb_12_ were also found from both theoretical and experimental approaches.^27–31^

Previous investigations also demonstrated that many pure clusters formed by 13 transition metal atoms in both neutral and ionic states exist in ideal or distorted icosahedral shape, including the first-row transition metal clusters such as Sc_13_, Ti_13_, V_13_, Cr_13_, Mn_13_ and Fe_13_ and heavier transition metal clusters such as Y_13_, Hg_13_, Zr_13_, Lu_13_ and Hf_13_.^32–34^ A characteristic difference of the latter from the main group element clusters is that most of the transition metal icosahedra possess very high total spin and thereby large magnetic moments, due to their unpaired *n*d electrons. Along with the homonuclear icosahedra, it was found that many doped transition metal clusters favor an icosahedral shape. Datta *et al.* reported from DFT calculations that the most stable isomers of vanadium doped cobalt clusters Co_13−*m*_V_*m*_, with *m* = 1–4, adopt an icosahedral geometry, unlike the hexagonal symmetry preference of the pure Co_13_ clusters.^35^ Other theoretical studies also demonstrated that an icosahedral 13-atom structure can be formed by endohedrally doping a transition metal atom into a coinage metal cluster Cu_12_ and Ag_12_.^36–39^ In particular, doping of a Cr atom into Cu_12_ as well as Ag_12_ was shown to form a Kondo-like system that enhances the thermodynamic stability of both resulting CrCu_12_ and CrAg_12_ icosahedra but quenches the large magnetic moment of the dopant simultaneously.^37,39^

Of the transition metal clusters possessing icosahedral geometry, the scandium-based clusters could deserve more attention because of their formal similarities with aluminum-based clusters in terms of valence electrons. As a matter of fact, the scandium element belongs to group IIIB and also has 3 valence electrons (3d^1^4s^2^). Additionally, both Sc_13_ and Al_13_ are characterized by a perfect icosahedral structure. However, unlike the anion Al_13_^−^ having a closed shell electronic structure and the neutral Al_13_ exhibiting a doublet ground state, both anionic and neutral states of Sc_13_ are known to exist in very high spin ground states, with the total spin magnetic moments of 18 and 19 *μ*_B_, respectively.^32,40^ A previous theoretical investigation of the singly aluminum doped scandium clusters Sc_*n*_Al, with *n* = 1–8, 12, showed that the Sc_*n*_Al isomers prefer an Al substitution at a Sc position of a structure of the corresponding Sc_*n*+1_ size. In fact, the resulting doped cluster Sc_12_Al exists in a high spin state along with an icosahedral shape in which the Al dopant is put into the center.^41^ Surprisingly, despite such a formal association between Sc and Al, an understanding of the geometric structure and electronic properties of the mixed Sc–Al clusters is still limited; they have not much been investigated so far. In this context, we set out to perform a detailed and systematic investigation on the binary Al–Sc clusters Al_*x*_Sc_*y*_ with *x* + *y* = 13, at both anionic and neutral states using DFT method, with the purpose of gaining deeper insights into the structural and electronic features of these intriguing systems. A question of interest is as to whether the icosahedral geometry, ideal or distorted, remains predominant following atomic mixture, and the identity of the central atom.

## 2. Computational methods

All standard electronic structure calculations in this study are carried out using the Gaussian 09 package.^42^ The possible isomers of each cluster are searched for using different approaches. In the first step, a stochastic genetic algorithm^43^ is used to generate the possible structures of each size Al_*x*_Sc_*y*_ with *x* + *y* = 13. This algorithm has been used in previous studies and shown to be highly efficient for systems containing different components.^18,44–46^ Another way of generating the initial isomers is a manual substitution by a Sc-atom at all Al positions of the well-known icosahedron Al_13_ to generate the Al_12_Sc structures, and successively for the following sizes with more Sc atoms. The search also includes the shapes of other 13-atom clusters that have previously been reported. Geometry optimizations are first carried out to generate the first initial set of isomers using the popular hybrid B3LYP functional in combination with the small LANL2DZ basis set.^47^ The local energy minima identified with relative energies of <5 eV with respect to the corresponding lowest-lying isomer of each size are then reoptimized at various spin states using the same functional but with the larger 6-311+G(d) basis set.^48^ Harmonic vibrational frequencies are subsequently calculated at this level to confirm the identity of the true local minima obtained, as well as to evaluate their zero-point energy corrections.

The lower-lying isomers of the neutral 13-atom clusters Al_*x*_Sc_*y*_ that have 39 valence electrons each, are then obtained from the corresponding anionic isomers upon removal of an electron and then geometrically optimized at different spin states. Moreover, a natural bond orbital (NBO) analysis is performed to examine the electronic configuration and thereby to rationalize the chemical bonding and magnetic properties of the clusters considered by using the NBO 3.1 program implemented in Gaussian 09. The total and local electron densities are defined as the difference between the numbers of spin-up and spin-down electrons occupying the molecular/atomic orbitals of the cluster/atom.

## 3. Results and discussion

### 3.1. Lower-lying isomers of binary Al–Sc clusters in both anionic and neutral states

**Fig. 1:**
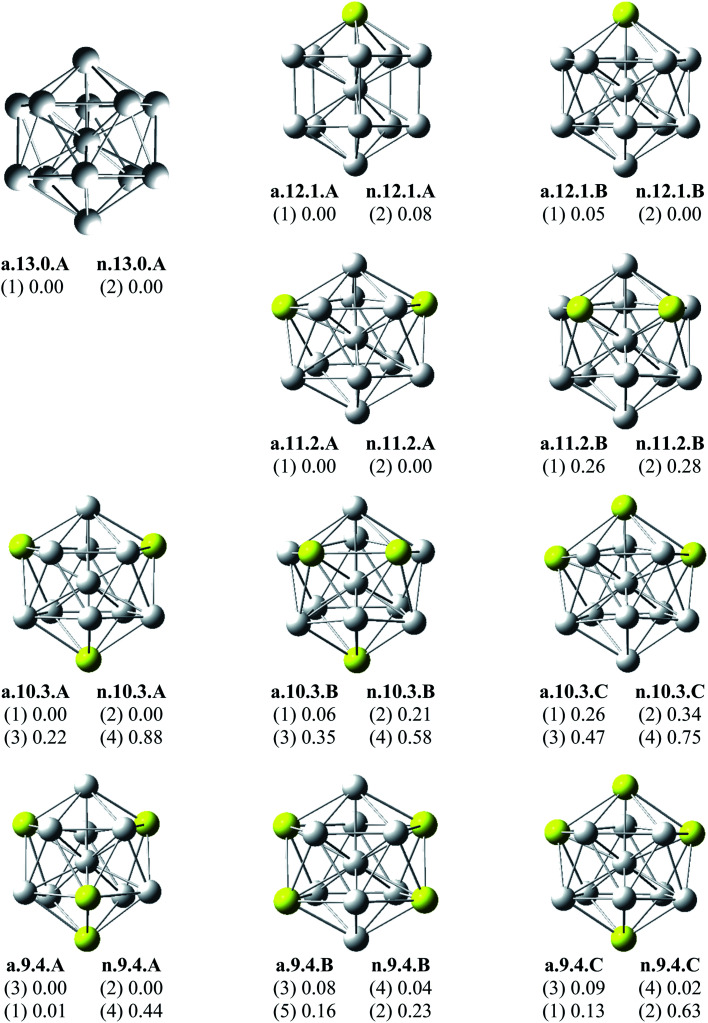
Geometry, relative energy (with ZPE corrections), and spin state (in the bracket) of the most stable isomers Al_*x*_Sc_*y*_ (*x* + *y* = 13 with *y* = 0–4) using (U)B3LYP/6-311+G(d) optimizations.

**Fig. 2:**
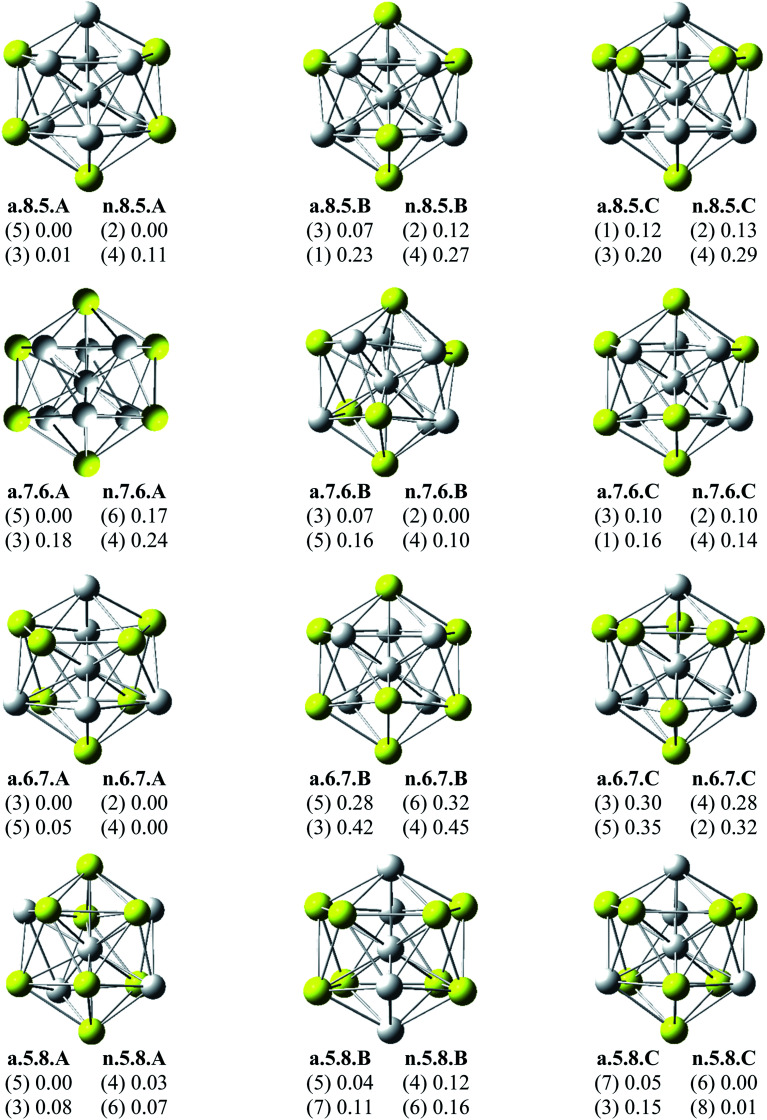
Geometry, relative energy (with ZPE corrections), and spin state (in the bracket) of the most stable isomers Al_*x*_Sc_*y*_ (*x* + *y* = 13 with *y* = 5–8) using (U)B3LYP/6-311+G(d) optimizations.

**Fig. 3:**
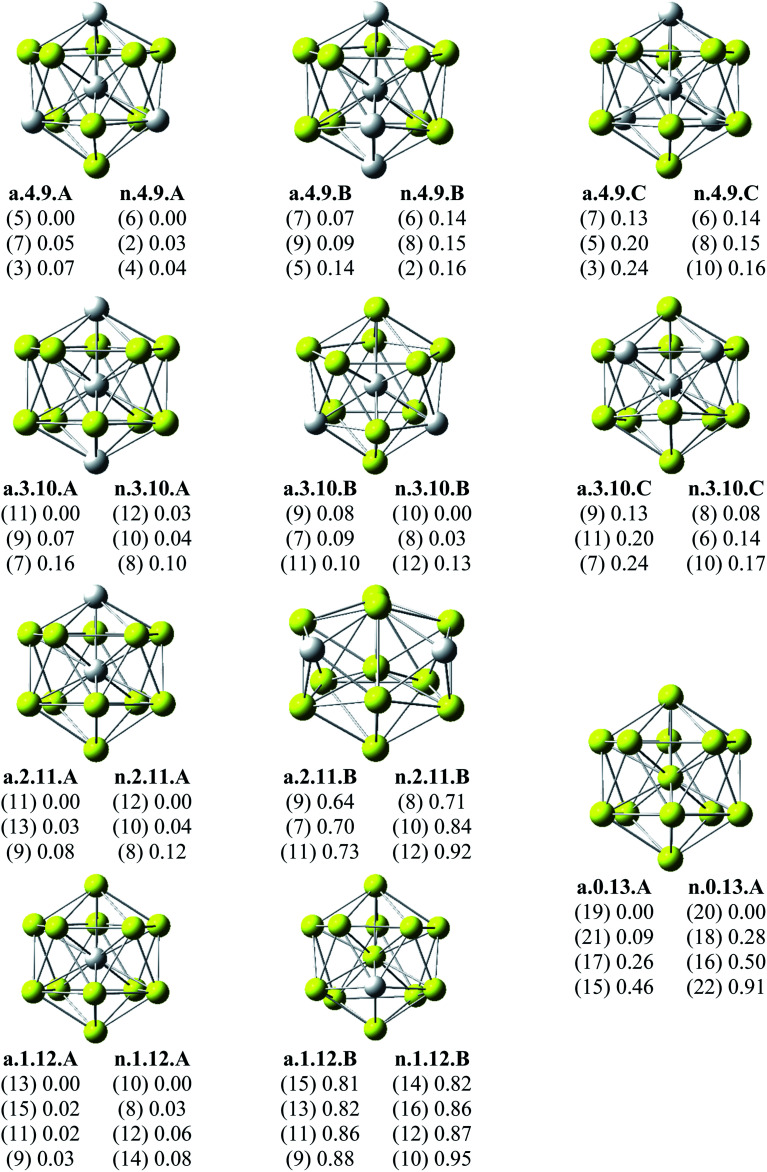
Geometry, relative energy (with ZPE corrections), and spin state (in the bracket) of the most stable isomers Al_*x*_Sc_*y*_ (*x* + *y* = 13 with *y* = 9–13) using (U)B3LYP/6-311+G(d) optimizations.

Since there is a large number of local minima located on the potential energy surface of each size considered in different spin states, we only present here the lowest-lying isomers whose relative energies are close to the corresponding most stable structure, being <1 eV in relative energy. The shapes of Al_*x*_Sc_*y*_ equilibrium structures in both anionic and neutral states, their spin states, and their relative energies obtained at the B3LYP/6-311+G(d)+ZPE level are shown in Fig. 1–3.

As for a convention, the S.*x*.*y*.Z label is used to denote the isomers in which S = a and n stand for an anionic state and its corresponding neutral with a similar geometrical shape, respectively, *x* being the number of Al atoms and *y* the number of Sc atoms, and Z = A, B, C… referring to the isomers with an increasing relative energy. Accordingly, the notation a.*x*.*y*.A invariably refers to the most stable anionic isomer (A) of the a.*x*.*y* series, and the n.*x*.*y*.A to its corresponding neutral.

Calculated results reveal an interesting discovery about geometrical features. All the lowest-lying Al_*x*_Sc_*y*_ isomers in both anionic and neutral states, irrespective of the number *x* of Al atoms, have an icosahedral shape in which an Al atom is invariably situated at the cage center, whereas the remaining Al and Sc atoms form the corresponding icosahedron in different positions.

For the size *x* = 12 Al_12_Sc^−^, a.12.1.A, formed by capping the Sc atom on a vertex of the bicapped pentagonal prism framework, is only 0.05 eV lower in energy than the icosahedral structure a.12.1.B in which the Sc atom is placed on a surface position of the icosahedron. However, such a relative energy gap is too small to be meaningful, and therefore both isomers can be considered as energetically degenerate. DFT results also point out some energetic degeneracies with small energy gaps of <0.1 eV for most of the (*x*, *y*) combination of the series of Al_*x*_Sc_*y*_ with *y* > 2. These isomers have an icosahedral framework with different positions of Sc atoms on the surface and in different spin states. As stated above, a remarkable feature of Al_*x*_Sc_*y*_ structures is that an Al atom is consistently found at the center regardless of the number of Al atoms. The fact that the Sc atoms occupy surface positions of the icosahedron can be understood by the smaller atomic radius of aluminum which favors the Al atom to occupy a position inside the cage. This is in agreement with a previous experimental study on the argon physisorption ability of the first row transition metals,^49^ showing that the transition metal doped clusters Al_*n*_TM^+^ are able to attach one argon atom up to a critical cluster size *n*_crit_, with *n*_crit_ = 16 for TM = V, Cr and *n*_crit_ = 19–21 for TM = Ti, and undergo a geometrical transition in going from exohedrally to endohedrally doped clusters in which the transition metal atom becomes, from *n*_crit_, located inside an aluminum cage. Furthermore, this feature can be rationalized on the basis of the strength of the homo- and heteronuclear bonds between Al and Sc atoms. According to DFT calculations, the bond energies of the Al_2_, AlSc and Sc_2_ dimers, also obtained at the B3LYP/6-311+G(d) level, amount to 1.2, 0.6, and 0.5 eV, respectively. Although bond energies of the dimers are expected to differ from the energies of corresponding bonds in the clusters, this result shows that in the cluster the bond between two Al atoms is much stronger than the Al–Sc and Sc–Sc bonds. In a Al_*x*_Sc_*y*_ cage structure both Al and Sc atoms are arranged in such a way of favoring formation of a maximum number of strong bonds, along with a minimum number of weak bonds. Accordingly, the Al atom thus prefers to occupy the icosahedral center in order to maximize the possible number of Al–Al and Al–Sc bonds.

### 3.2. Electron configuration and multiplicity

**Fig. 4:**
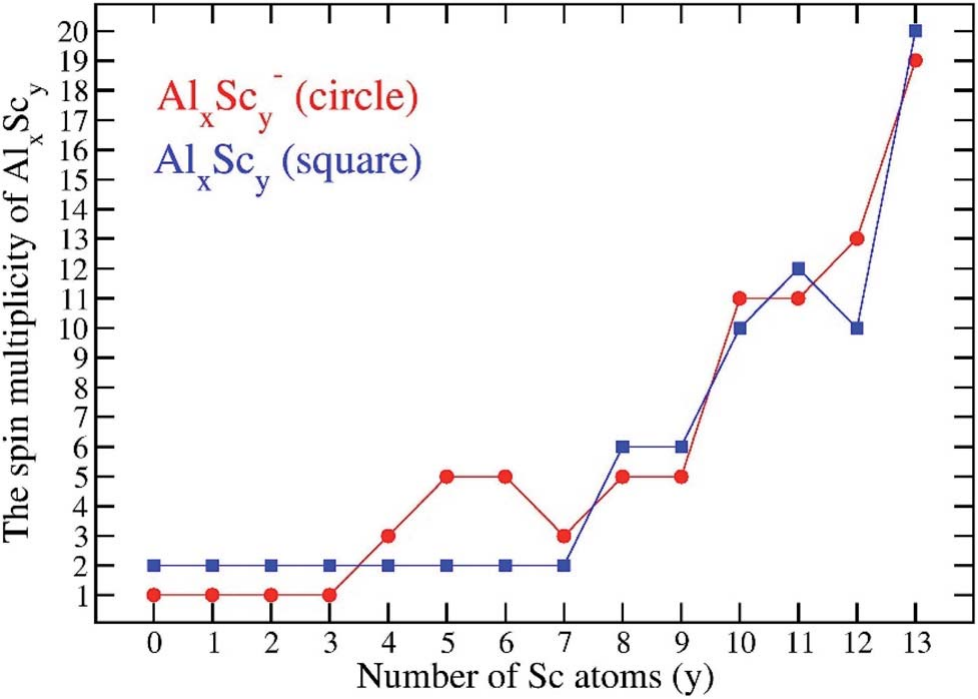
Spin multiplicities of the most stable isomers of the binary Al_*x*_Sc_*y*_ clusters considered.

Along with a consistent possession of an icosahedral shape, another typical feature is that the Al_*x*_Sc_*y*_ ground electronic states are associated with high number of unpaired electrons. The search for the most stable isomers is carried out for all plausible electron spin state for each cluster composition, and the multiplicity of Al_*x*_Sc_*y*_ tends to gradually increase with an increasing number of Sc atoms. For a small number of Sc atoms, *y* = 0–3, the most stable structures of Al_12_Sc, Al_11_Sc_2_ and Al_10_Sc_3_ and their anions keep the lowest spin states alike the Al_13_ cluster, that are singlet state for species having an even number of electrons and doublet state for those having an odd number of electrons. A competition between the low and high spin states begins to occur at the size of four Sc atoms, namely the Al_9_Sc_4_. For *y* = 10–13, both anionic and neutral Al_*x*_Sc_*y*_ structures are more stable at very high spin state in which each has nine or more unpaired electrons, approaching that of the pure scandium Sc_13_. Indeed, the most stable isomer of the latter has the highest spin states with 18 and 19 open shells for both anionic and neutral states, respectively. Fig. 4 displays the variation of the multiplicity of the binary clusters considered.

In order to rationalize the high spin states of Al_*x*_Sc_*y*_ clusters and obtain a deeper understanding of their electronic structures, we now analyze the natural bond orbitals of the anions.

**Fig. 5:**
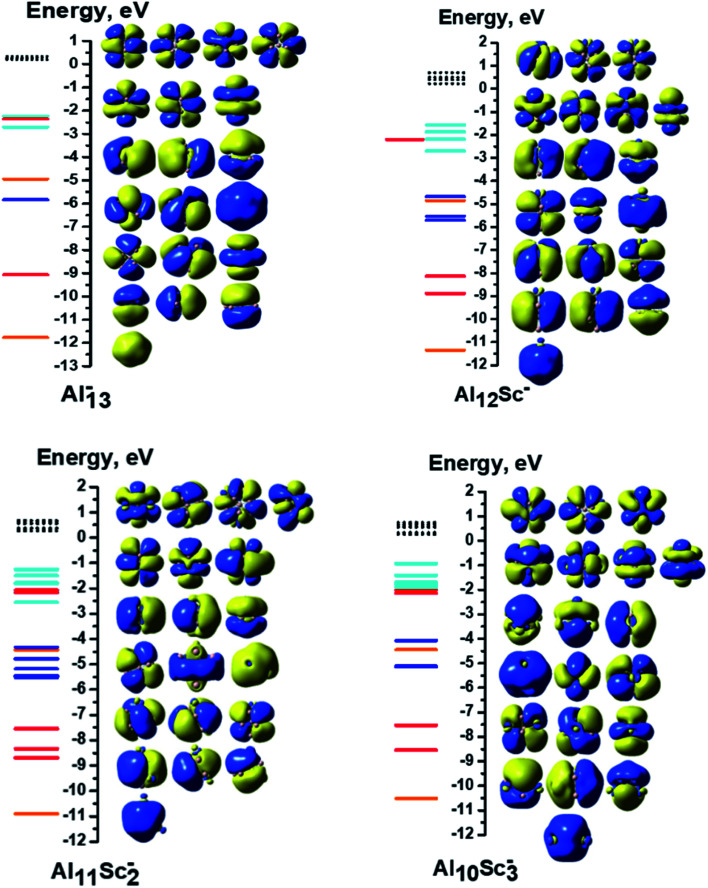
MO interaction diagrams of the anions Al_*x*_Sc_*y*_^−^ (*x* + *y* = 13 with *y* = 0–3) with shapes of the delocalized orbitals and the localized 3d Sc atomic orbitals. The orange, red, blue, cyan lines represent orbitals corresponding to the 1S, 1P, 1D and 1F cluster shells, respectively. The green lines represent the localized 3d AO(Sc)s. The filled lines stand for occupied orbitals and the dashed lines denote the unoccupied ones.

Each 13-atom Al_*x*_Sc_*y*_^−^ anion has 40 valence electrons, as each of the Al and Sc atoms has three valence electrons. For Al_13_^−^, all of its 40 valence electrons contribute to the shell molecular orbitals resulting in a closed electron shell of [1S^2^1P^6^1D^10^2S^2^2P^6^1F^14^], without any unpaired electron, as shown in Fig. 5. Similarly, the Al_12_Sc^−^, Al_11_Sc_2_^−^, and Al_10_Sc_3_^−^ anions also have the same closed electron configuration of [1S^2^1P^6^1D^10^2S^2^2P^6^1F^14^] without open shell. The clusters having up to three Sc atoms do not have any Sc–Sc bond for the reason described above, as their geometrical structures seen in Fig. 1. In these cases, the isolation of Sc atoms around an Al environment results in a quenching of its spin upon formation of the Al–Sc bonds. In order to understand how the Al_*x*_Sc_*y*_ clusters come to possess high multiplicity upon increase of the Sc atom number going from four up to thirteen, we would start examining the electron configuration of the pure Sc_13_ cluster which is characterized by the highest spin multiplicity as a special reference case, and the other binary clusters with a reduced number of Sc atoms, going down to Al_9_Sc_4_, are subsequently examined.

#### 3.2.1. The Sc_13_^−^ cluster

**Fig. 6:**
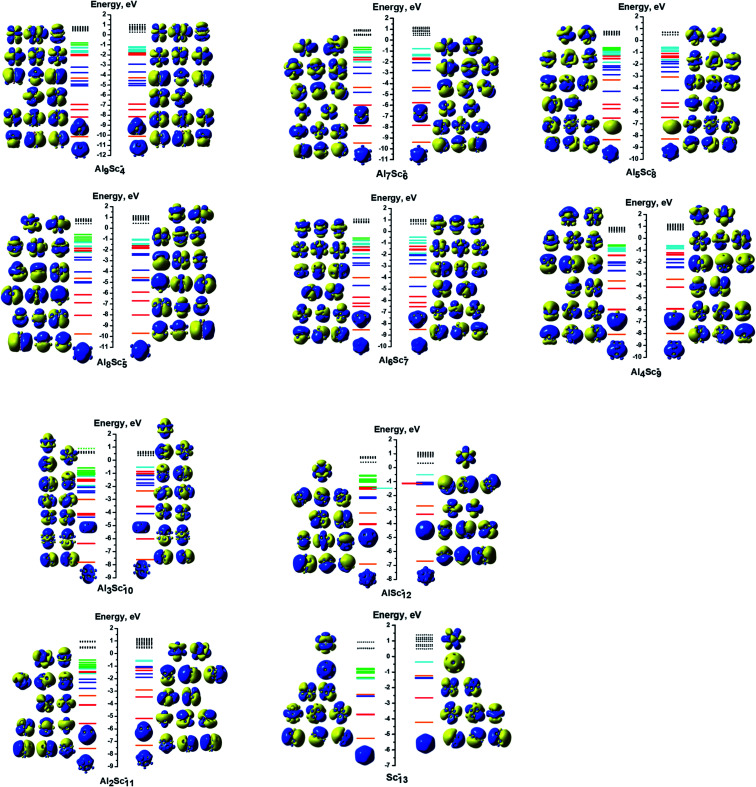
MO interaction diagrams of the anions Al_*x*_Sc_*y*_^−^ (*x* + *y* = 13 with *y* = 4–13) with shapes of the delocalized orbitals and the localized 3d Sc atomic orbitals. The orange, red, blue, cyan lines represent orbitals corresponding to the 1S, 1P, 1D and 1F cluster shells, respectively. The green lines represent the localized 3d AO(Sc)s. The filled lines stand for occupied orbitals and the dashed lines denote the unoccupied ones.

In contrast to the closed electron shell of Al_13_^−^, and in line with the previous investigations,^32–34^ despite having 40 valence electrons, the anionic icosahedron Sc_13_^−^ possesses open electron shells containing 18 unpaired electrons, and therefore only 22 valence electrons are filling its shell orbitals. The electron configuration of the Sc_13_^−^ anion can be written as [1S^2^1P^6^1D^10^2S^2^1F^2^SOMO^18^], as shown in Fig. 6. From the atomic orbital (AO) contributions to the singly occupied molecular orbitals (SOMO) of Sc_13_^−^, displayed in Table 1, the 18 SOMOs of Sc_13_^−^ are composed of 1AOs, 2AOp and 15AOd of 13 Sc atoms. Of the 18 unpaired electrons, 1.4–1.5 electron is distributed on each of the surrounding Sc atoms, whereas only ∼0.5 electron stays on the central Sc atom (*cf.*Table 2). There is thus a good agreement with the results reported by Gutsev *et al.*^32^ as these authors found that each exohedral Sc atom has ∼1.5 unpaired electron and the central Sc barely a charge of ∼−0.2 electron.

**Table:** AOs contributions in the SOMOs of the anions Al_*x*_Sc_*y*_^−^

Clusters	Sc					Al	
	4s	4p	3d	4d	5d	3s	3p
Sc_13_^−^	0.99	1.77	14.81	0.25	0.00	—	—
AlSc_12_^−^	−0.05	0.01	11.84	0.13	0.00	−0.02	−0.01
Al_2_Sc_11_^−^	0.04	0.44	9.20	0.11	0.00	0.00	0.15
Al_3_Sc_10_^−^	−0.04	0.31	9.23	0.19	0.00	0.00	0.34
Al_4_Sc_9_^−^	0.12	0.32	3.65	0.06	0.00	−0.02	−0.2
Al_5_Sc_8_^−^	0.03	0.25	3.55	0.06	0.00	0.02	0.05
Al_6_Sc_7_^−^	−0.06	0.12	2.37	0.05	0.00	−0.12	−0.41
Al_7_Sc_6_^−^	−0.14	0.10	3.97	0.10	0.00	−0.10	0.03
Al_8_Sc_5_^−^	0.05	0.33	2.99	0.07	0.00	0.09	0.42
Al_9_Sc_4_^−^	0.27	0.23	1.24	0.02	0.00	0.08	0.12

Local and total unpaired electrons of anionic Al_*x*_Sc_*y*_^−^ clusters

**Table:** 

No.	Al_9_Sc_4_^−^		Al_8_Sc_5_^−^		Al_7_Sc_6_^−^		Al_6_Sc_7_^−^		Al_5_Sc_8_^−^	
1	Al	0.1	Al	0.1	Al	0.0	Al^a^	0.0	Al	0.1
2	Al^a^	0.0	Al^a^	0.0	Al	0.0	Al	0.0	Al	0.1
3	Al	0.1	Al	0.1	Al^a^	0.0	Al	0.0	Al	0.0
4	Al	0.1	Al	0.1	Al	0.0	Al	−0.1	Al^a^	0.0
5	Al	0.1	Al	0.1	Al	0.0	Al	−0.2	Al	0.0
6	Al	0.0	Al	0.1	Al	0.0	Al	−0.2	Sc	0.4
7	Al	0.0	Al	0.1	Al	0.0	Sc	0.2	Sc	0.4
8	Al	0.0	Al	0.0	Sc	0.7	Sc	0.5	Sc	0.6
9	Al	0.0	Sc	0.8	Sc	0.7	Sc	0.6	Sc	0.3
10	Sc	0.8	Sc	0.8	Sc	0.7	Sc	0.5	Sc	0.7
11	Sc	0.0	Sc	0.7	Sc	0.7	Sc	0.2	Sc	0.6
12	Sc	0.8	Sc	0.7	Sc	0.7	Sc	0.0	Sc	0.7
13	Sc	0.0	Sc	0.4	Sc	0.7	Sc	0.6	Sc	0.3
	Total	2.0	Total	4.0	Total	4.0	Total	2.0	Total	4.0

a The atom locates at center of icosahedral cage.

**Table:** 

No.	Al_4_Sc_9_^−^		Al_3_Sc_10_^−^		Al_2_Sc_11_^−^		AlSc_12_^−^		Sc_13_^−^	
1	Al	0.0	Al	0.2	Al^a^	0.0	Al^a^	0.0	Sc	1.4
2	Al^a^	0.0	Al^a^	0.0	Al	0.2	Sc	1.0	Sc	1.5
3	Al	0.0	Al	0.2	Sc	0.9	Sc	1.0	Sc	1.4
4	Al	0.0	Sc	0.9	Sc	0.8	Sc	1.0	Sc	1.4
5	Sc	0.3	Sc	1.1	Sc	0.8	Sc	1.0	Sc	1.5
6	Sc	0.3	Sc	0.9	Sc	1.0	Sc	1.0	Sc	1.5
7	Sc	0.1	Sc	1.0	Sc	1.0	Sc	1.0	Sc	1.4
8	Sc	0.6	Sc	1.1	Sc	0.9	Sc	1.0	Sc	1.5
9	Sc	0.7	Sc	0.9	Sc	0.9	Sc	1.0	Sc^a^	0.5
10	Sc	0.6	Sc	1.0	Sc	0.9	Sc	1.0	Sc	1.4
11	Sc	0.5	Sc	1.0	Sc	0.9	Sc	1.0	Sc	1.4
12	Sc	0.5	Sc	0.9	Sc	0.9	Sc	1.0	Sc	1.5
13	Sc	0.8	Sc	1.0	Sc	0.9	Sc	1.0	Sc	1.5
	Total	4.0	Total	10	Total	10	Total	12	Total	18

Moreover, an observation of the shapes of 18 SOMOs points out that they are neither the shell orbitals (such as 1F, 2D…) of the Sc_13_^−^ cluster nor the individual AOs of any Sc atom but rather they are the molecular orbitals that belong to the A, E and T irreducible representations of a *T* point group. Accordingly, the electron configuration of the Sc_13_^−^ cluster can best be written as [1S^2^1P^6^1D^10^2S^2^1F^2^3d_E_^2^3d_T_^3^3d_A_^1^3d_E_^2^3d_A_^1^3d_T_^3^3d_A_^1^3d_A_^1^3d_E_^2^3d_E_^2^]. In this electron configuration, the notations of 3d_A_, 3d_E_ and 3d_T_ represent the SOMOs that are formed from combinations of 3d atomic orbitals of Sc atoms and belong to the A, E and T irreducible representations of *T* point group, respectively.

#### 3.2.2. The AlSc_12_^−^ cluster

The most stable isomer of the AlSc_12_^−^ anion is characterized by twelve (12) open shells, corresponding to a 13-et multiplicity. Therefore, 28 valence electrons fill the cluster shell orbitals resulting from an electron configuration of [1S^2^1P^6^1D^10^2S^2^2P^6^1F^2^SOMO^12^] (*cf.*Fig. 6). Table 2 shows that no unpaired electron is placed on the central Al atom of the icosahedral cage of AlSc_12_^−^.

A similar phenomenon occurs in the remaining Al_*x*_Sc_*y*_^−^ sizes with a decreasing Al atom number. On average, each dAO(Sc) of AlSc_12_^−^ contains 0.2 unpaired electron, proportionately 1.0 unpaired electron found on each Sc atom. Thus, those twelve spatial orbitals are no longer individual 3d orbitals of any single Sc atom, but rather their combination creates molecular orbitals that belong to the A, E and T irreducible representations of the *T* point group. Similar to the previous case, the electron configuration of the Al_1_Sc_12_^−^ cluster can be written as [1S^2^1P^6^1D^10^2S^2^2P^6^1F^2^3d_E_^2^3d_A_^1^3d_E_^2^3d_A_^1^3d_E_^2^3d_E_^2^3d_E_^2^].

#### 3.2.3. The Al_2_Sc_11_^−^ cluster

The Al_2_Sc_11_^−^ ground state has 10 unpaired electrons, corresponding to an 11-et multiplicity. Its 30 valence electrons fill the shell orbitals resulting the electron configuration of [1S^2^1P^6^1D^10^2S^2^2P^6^1F^4^SOMO^10^]. The 10 unpaired electrons occupy the d orbitals of all 11 Sc atoms. This is confirmed by the results of the local and total unpaired electrons derived from the NBO calculation, as represented in Table 2. Again, the ten SOMOs combination lead to MOs that belong to the A and E irreducible representations of a *C*_5V_ point group. The electron configuration of the Al_2_Sc_11_^−^ anion can be expressed as [1S^2^1P^6^1D^10^2S^2^2P^6^1F^4^3d_E_^2^3d_A_^1^3d_E_^2^3d_E_^2^3d_E_^2^3d_A_^1^].

Similarly, the electron configuration based on the symmetry point group and spin multiplicity of the remaining anions can concisely be expressed in the same way as follows:

The Al_3_Sc_10_^−^ cluster (*C*_5v_, 11-et). Each of the Sc atoms contains one unpaired electron and 30 valence electrons are paired in the shell orbitals of the electron configuration [1S^2^1P^6^1D^10^2S^2^2P^6^1F^4^3d_E_^2^3d_E_^2^3d_A_^1^3d_E_^2^3d_E_^2^3d_A_^1^].

The Al_4_Sc_9_^−^ cluster (*C*_3v_, quintet). Its electron configuration can be written as [1S^2^1P^6^1D^10^2S^2^2P^6^1F^10^3d_E_^2^3d_A_^1^3d_A_^1^].

The Al_5_Sc_8_^−^ cluster (*C*_2_, quintet). The ordering of its valence electron filling is [1S^2^1P^6^1D^10^2S^2^2P^6^1F^10^3d_A_^1^3d_B_^1^3d_A_^1^3d_B_^1^].

The Al_6_Sc_7_^−^ cluster (*C*_s_, triplet). The remaining 38 valence electrons are coupled in pairs in the electron shells of [1S^2^1P^6^1D^10^2S^2^2P^6^1F^12^3d_A′′_^1^3d_A′_^1^].

The Al_7_Sc_6_^−^ cluster (*C*_3v_, quintet). The filling of valence electrons of this cluster is [1S^2^1P^6^1D^10^2S^2^2P^6^1F^10^3d_A_^1^3d_E_^2^3d_A_^1^].

The Al_8_Sc_5_^−^ cluster (*C*_s_, quintet) has an electron configuration of [1S^2^1P^6^1D^10^2S^2^2P^6^1F^10^3d_A′′_^1^3d_A′′_^1^3d_A′_^1^3d_A′_^1^].

The Al_9_Sc_4_^−^ cluster (*C*_2v_, triplet). The 38 paired valence electrons fill the shell orbitals resulting in an electron configuration of [1S^2^1P^6^1D^10^2S^2^2P^6^1F^12^3d_A1_^1^3d_A1_^1^].

The symmetries, spin multiplicities, and electron configurations of all anionic Al_*x*_Sc_*y*_^−^ clusters are also summarized in Table 4. In general, in the open shell systems of Al_*x*_Sc_*y*_^−^, with *y* = 4–13, the unpaired electrons are mostly distributed on the Sc atoms located at the cage vertexes. Moreover, the spatial orbitals of the SOMOs are neither the shell molecular orbitals nor the individual AOs of any Sc atoms of the Al_*x*_Sc_*y*_^−^ clusters, but rather the MOs that belong to the irreducible representations of the corresponding point group, depending on the geometrical symmetry of the cluster considered.

As shown in Table 4, the calculation results show that all the d electrons of Sc atoms take part in cluster bonding in the Al_12_Sc^−^, Al_11_Sc_2_^−^, and Al_10_Sc_3_^−^ clusters while the Al_*x*_Sc_*y*_^−^ clusters contain unpaired d electrons when *x* is larger than three. In the clusters Al_12_Sc^−^, Al_11_Sc_2_^−^, and Al_10_Sc_3_^−^ the Sc atoms are far apart from each other, their d electrons rather participate in formation of bonds with the Al atoms. For the Al_*x*_Sc_*y*_^−^ (*x* > 3) clusters, the Sc atoms are located next to each other (see Fig. 1 and 2) and their 3d unpaired electrons are not bonded but having parallel spins that create a magnetism.

In the cluster Al_9_Sc_4_^−^, each of the Sc^(10)^ and Sc^(12)^ atoms, which are next to each other, has 0.8 unpaired electrons and these unpaired electrons mainly belong to their d AOs (see Tables 1 and 2). Therefore, it can be concluded that they do not use their single electron d AOs to form bonds with each other.

In the cluster Al_8_Sc_5_^−^, the Sc^(9)^, Sc^(10)^ atoms are the neighbors of Sc^(12)^ and Sc^(11)^ ones, respectively, at a distance of 2.970 Å, 0.148 Å closer than the distance to the Sc^(13)^ atom. Tables 1 and 2 show that each of the Sc^(9)^, Sc^(10)^, Sc^(11)^ and Sc^(12)^ atoms have ∼0.75 single electrons that mainly belong to their d AOs. Therefore, we can also conclude that these Sc atoms do not use their unpaired d electron to form bonds with each other.

In the Al_7_Sc_6_, the six Sc atoms next to each other forming a ring with equal Sc–Sc distance of 3.043 Å. Tables 1 and 2 show that each Sc atom has ∼0.7 unpaired electrons and these unpaired electrons mainly belong to their d AOs.

In the case of Al_6_Sc_7_^−^, Al_5_Sc_8_^−^, we observe the same phenomenon as in previous clusters having four or more Sc atoms. Although the number of Sc atoms increases, there are still enough Al atoms in the alternative positions of the Sc atoms and their magnetism does not increase. A leap in the magnetic property is observed in subsequent clusters from Al_3_Sc_10_^−^, Al_2_Sc_11_^−^ to Al_1_Sc_12_^−^, in which the number of Al atoms becomes sufficiently small that all the Sc atoms are located in positions next to each other with similar distance and do not use their d AOs for bonding to each other. Therefore, they exhibit 10, 10 and 12 unpaired electrons, respectively.

A special case involves the Sc_13_^−^ where no Al atom is present and all Sc atoms do not use their d AOs, and some of their s and p AOs for bonding. As a result, the cluster is exceptionally magnetic, bearing up to 18 unpaired electrons.

### 3.3. Thermodynamic stability

The inherent thermodynamic stability of the 13-atom clusters Al_*x*_Sc_*y*_ are now evaluated through the examination of average binding energies (*E*_b_) which can conventionally be defined in eqn (1) and (2):

1 *E*_b_(Al_*x*_Sc_*y*_^−^) = [(*x* − 1)*E*(Al) + *E*(Al^−^) + *yE*(Sc) − *E*(Al_*x*_Sc_*y*_^−^)]/13

2 *E*_b_(Al_*x*_Sc_*y*_) = [*xE*(Al) + *yE*(Sc) − *E*(Al_*x*_Sc_*y*_)]/13

**Fig. 7:**
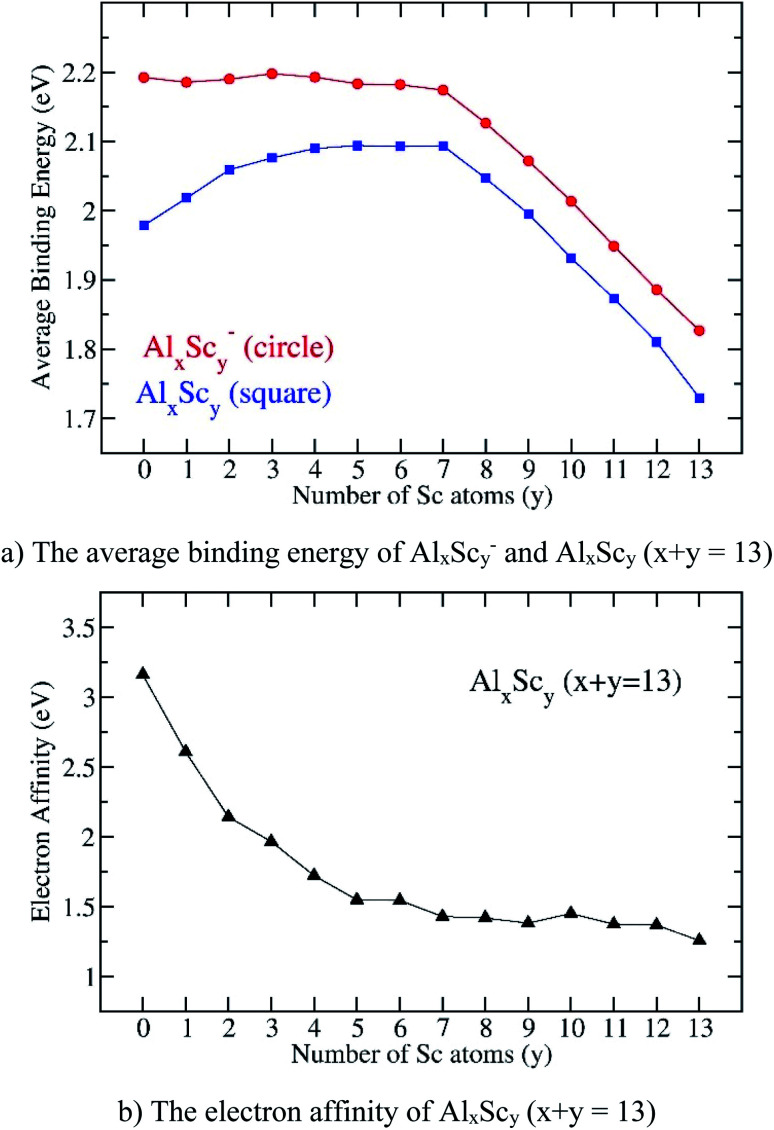
Evolution of (a) the average binding energy and (b) electron affinity of the Al_*x*_Sc_*y*_ clusters considered in their ground state. Values are obtained from (U)B3LYP/6-311+G(d)+ZPE computations.

Particularly for the anionic Sc_13_^−^, the *E*_b_ can be defined by eqn (3):

3 *E*_b_(Sc_13_^−^) = [12*E*(Sc) + *E*(Sc^−^) − *E*(Sc_13_^−^)]/13

where *E*(Al), *E*(Al^−^), *E*(Sc), *E*(Sc^−^), *E*(Al_*x*_Sc_*y*_), and *E*(Al_*x*_Sc_*y*_^−^) are the total energies of the Al-atom, the anion Al^−^, the Sc-atom, the anion Sc^−^, the neutral Al_*x*_Sc_*y*_, and the anion Al_*x*_Sc_*y*_^−^, respectively. Since the electron affinity of the Al atom (EA(Al) = 0.43 eV)^50^ is larger than that of the Sc atom (EA(Sc) = 0.19 eV),^51^ the total energy of the anion Al^−^ is thus used to calculate the average binding energy instead of that of the anion Sc^−^. All these energies are obtained from DFT calculations and the plots of *E*_b_ depicted in Fig. 7a illustrate their evolution.

There is no significant change of *E*_b_ values of the anions Al_*x*_Sc_*y*_^−^ whereas those values of the neutral structures increase when *y* goes from 0 to 4. For *y* = 4–7, the *E*_b_ values of the anions slightly decrease whereas those of neutrals continue to increase, even slightly. However, when the Sc atom number is greater than seven, the *E*_b_ of both anionic and neutral states steadily decrease, and attains the lowest value at *y* = 13, corresponding to the value of the pure scandium cluster Sc_13_. In order to interpret such a trend, the total Al–Al, Al–Sc and Sc–Sc bond order within each cluster are analyzed using NBO calculations.

The bond order for each of the bonds connecting two atoms, including Al–Al, Al–Sc, and Sc–Sc in the clusters can be calculated as half of the difference between the electron occupancies in the corresponding bonding and anti-bonding orbitals. The total bond order for each of the bonds involved are thus summed up in the total Al–Al, Al–Sc and Sc–Sc bond orders. The values of bond orders, NBO charges of the central atom of the icosahedral cage are listed in the Table 3. In going from Al_13_^−^ to Al_7_Sc_6_^−^, the Al–Al bond order decreases, whereas the Al–Sc and Sc–Sc bond orders increase, and the total bond order of each cluster remains relatively high. Therefore, the energy needed to break all the bonds in a cluster to form the constituent atoms is high as compared to that in other clusters. It is worth noting again that the Al–Al bond is markedly stronger than those made between Sc and Sc (*cf.* above). Nevertheless, the Sc–Sc bond order in the Sc_2_ dimer amounts to 2.3, being much larger as compared to that of the Al–Al dimer.^52^ From the size Al_6_Sc_7_^−^ onward to AlSc_12_^−^ and finally Sc_13_^−^, the Al–Al bond order of Al_*x*_Sc_*y*_ is going close to zero, and this makes their average binding energies much lower as compared to those the Al_*x*_Sc_*y*_ clusters having *y* < 7. Consequently, the average binding energy tends to decrease from Al_6_Sc_7_^−^ to Al_1_Sc_12_^−^ and to Sc_13_^−^ and this could attribute to a substantial decrease in the Al–Sc bond order in these sizes.

**Table:** Summation of Al–Al, Al–Sc, Sc–Sc bond orders, total bond order and net charge of Al central atom in the anionic Al_*x*_Sc_*y*_^−^ clusters

Cluster	Summation of all Al–Al bond order	Summation of all Al–Sc bond order	Summation of all Sc–Sc bond order	Total bond order	Charge of center atom^a^	Charge of the remain cage
Al_13_^−^	7.3	0.0	0.0	7.3	−1.7	+0.7
Al_12_Sc_1_^−^	6.2	3.5	0.0	9.7	−1.1	+0.1
Al_11_Sc_2_^−^	3.6	7.1	0.0	10.7	−0.7	−0.3
Al_10_Sc_3_^−^	1.6	10.0	0.0	11.6	−0.4	−0.6
Al_9_Sc_4_^−^	2.5	4.6	0.8	7.8	−0.1	−0.9
Al_8_Sc_5_^−^	2.5	4.0	1.8	8.3	0.1	−1.1
Al_7_Sc_6_^−^	1.8	5.2	2.5	9.6	0.4	−1.4
Al_6_Sc_7_^−^	0.0	5.6	2.5	8.1	0.6	−1.6
Al_5_Sc_8_^−^	0.0	3.2	4.3	7.6	0.7	−1.7
Al_4_Sc_9_^−^	0.0	4.1	4.3	8.4	0.7	−1.7
Al_3_Sc_10_^−^	0.1	0.2	5.6	6.0	0.8	−1.8
Al_2_Sc_11_^−^	0.2	0.5	6.3	7.0	0.7	−1.7
Al_1_Sc_12_^−^	0.0	0.0	8.7	8.7	0.7	−1.7
Sc_13_^−^	0.0	0.0	8.9	8.9	−3.7	+2.7

a Except for Sc_13_^−^, all remaining clusters have Al atom located at the central position of the icosahedral cage.

**Table:** Point group, electron spin multiplicity and order of valence electron filling the shell MOs and the symmetric MOs from low to high energy level in Al_*x*_Sc_*y*_^−^ clusters. MO having unpaired electrons is marked in bold

Cluster	Point group	Spin multiplicity	Order of valence electron filling
Al_13_^−^	*I*_h_	1	1S^2^1P^6^1D^10^2S^2^2P^6^1F^14^
Al_12_Sc^−^	*C*_5V_	1	1S^2^1P^6^1D^10^2S^2^2P^6^1F^14^
Al_11_Sc_2_^−^	*C*_2V_	1	1S^2^1P^6^1D^10^2S^2^2P^6^1F^14^
Al_10_Sc_3_^−^	*C*_3V_	1	1S^2^1P^6^1D^10^2S^2^2P^6^1F^14^
Al_9_Sc_4_^−^	*C*_2V_	3	1S^2^1P^6^1D^10^2S^2^2P^6^1F^12^**3d**_**A1**_^**1**^**3d**_**A1**_^**1**^
Al_8_Sc_5_^−^	*C*_S_	5	1S^2^1P^6^1D^10^2S^2^2P^6^1F^10^**3d**_**A′′**_^**1**^**3d**_**A′′**_^**1**^**3d**_**A′**_^**1**^**3d**_**A′**_^**1**^
Al_7_Sc_6_^−^	*C*_3V_	5	1S^2^1P^6^1D^10^2S^2^2P^6^1F^10^**3d**_**A**_^**1**^**3d**_**E**_^**2**^**3d**_**A**_^**1**^
Al_6_Sc_7_^−^	*C*_S_	3	1S^2^1P^6^1D^10^2S^2^2P^6^1F^12^**3d**_**A′′**_^**1**^**3d**_**A′**_^**1**^
Al_5_Sc_8_^−^	*C*_2_	5	1S^2^1P^6^1D^10^2S^2^2P^6^1F^10^**3d**_**A**_^**1**^**3d**_**B**_^**1**^**3d**_**A**_^**1**^**3d**_**B**_^**1**^
Al_4_Sc_9_^−^	*C*_3V_	5	1S^2^1P^6^1D^10^2S^2^2P^6^1F^10^**3d**_**E**_^**2**^**3d**_**A**_^**1**^**3d**_**A**_^**1**^
Al_3_Sc_10_^−^	*C*_5V_	11	1S^2^1P^6^1D^10^2S^2^2P^6^1F^4^**3d**_**E**_^**2**^**3d**_**E**_^**2**^**3d**_**A**_^**1**^**3d**_**E**_^**2**^**3d**_**E**_^**2**^**3d**_**A**_^**1**^
Al_2_Sc_11_^−^	*C*_5V_	11	1S^2^1P^6^1D^10^2S^2^2P^6^1F^4^**3d**_**E**_^**2**^**3d**_**A**_^**1**^**3d**_**E**_^**2**^**3d**_**E**_^**2**^**3d**_**E**_^**2**^**3d**_**A**_^**1**^
Al_1_Sc_12_^−^	*T*	13	1S^2^1P^6^1D^10^2S^2^2P^6^1F^2^**3d**_**E**_^**2**^**3d**_**A**_^**1**^**3d**_**E**_^**2**^**3d**_**A**_^**1**^**3d**_**E**_^**2**^**3d**_**E**_^**2**^**3d**_**E**_^**2**^
Sc_13_^−^	*T*	19	1S^2^1P^6^1D^10^2S^2^1F^2^**3d**_**E**_^**2**^**3d**_**T**_^**3**^**3d**_**A**_^**1**^**3d**_**E**_^**2**^**3d**_**A**_^**1**^**3d**_**T**_^**3**^**3d**_**A**_^**1**^**3d**_**A**_^**1**^**3d**_**E**_^**2**^**3d**_**E**_^**2**^

Fig. 7a also shows the *E*_b_ values of all anionic Al_*x*_Sc_*y*_^−^ clusters that are obviously higher than those of the neutral counterparts, and this proves that the neutral tends to receive an electron to form the more stable anionic state. This feature can be observed more closely by examining the computed electron affinities shown in Fig. 7b. Starting from the superhalogen Al_13_ with a very large electron affinity, EA(Al_13_) = 3.2 eV, the substitution of one to five Al atoms in Al_13_ by a corresponding number of Sc atoms rapidly reduces this parameter, down to a value of 1.6 eV at Al_8_Sc_5_. When the Sc atom number increases from 5 to 13, the electron affinity turns out to slightly decrease and takes the lowest value of 1.3 eV at the pure Sc_13_. Such a reduction is no doubt due to the low electron affinity of the transition metal atom. Thus, substitution of Al atoms in Al_13_ by Sc atoms makes the binary clusters losing their superhalogen characteristic.

## 4. Concluding remarks

In the present theoretical study, the geometrical and electronic structures of the 13-atom clusters Al_*x*_Sc_*y*_, with *x* + *y* = 13, were investigated using DFT calculations. Geometry optimizations remarkably pointed out that all the most stable isomers of Al_*x*_Sc_*y*_, in both anionic and neutral states, retain the icosahedral shape in which the Al atom is favored to occupy the central location, irrespective of the Al atom number. The Sc atoms are consistently located at the vertexes of an icosahedral cage. The icosahedral shape is thus retained, even with some slight geometric distortions. The perfect icosahedral shape is kept only for the Al_13_, Sc_13_ and AlSc_12_ sizes.

The electron configurations of the clusters considered, in their ground state, have been established and rationalized along with the spin multiplicities. NBO analyses revealed that the unpaired electrons are mostly distributed on the Sc atoms and the SOMOs are the molecular orbitals belonging to the irreducible representations of the symmetry point group of the corresponding cluster. Thermodynamic stabilities were also examined through the average binding energy per atom in each size. The stable geometrical structure, the unpaired electron and thereby multiplicity, and the average binding energy follow some clear trends as follows:

(i) The scandium atoms prefer to be located on exohedral sites of icosahedron nearby the aluminum atoms in order to maximize the number of stronger Al–Al and Sc–Al bonds at the expense of the weaker Sc–Sc bonds;

(ii) The Al_*x*_Sc_*y*_ clusters are stable at low multiplicity when the Sc atom number goes from 0 to 3, whereas a high spin state predominates as the Sc atom number increases from 4 to 13, and

(iii) The cluster stability insignificantly changes from the superhalogen Al_13_ to the Al_6_Sc_7_ and then regularly decreases from the size Al_6_Sc_7_ to the pure Sc_13_. Moreover, substitution of Al atoms in Al_13_ by Sc atoms results in a loss of the superhalogen characteristics, in which the electron affinities of the binary Al_*x*_Sc_*y*_ superatoms are reduced with respect to that of the pure Al_13_ cluster.

## Conflicts of interest

The authors declare no competing financial interest.

## Supplementary Material
