# Correction: The binary aluminum scandium clusters Al_*x*_Sc_*y*_ with *x* + *y* = 13: when is the icosahedron retained?

**DOI:** 10.1039/d1ra90178h

**Published:** 2021-12-24

**Authors:** Ngo Tuan Cuong, Nguyen Thi Mai, Nguyen Thanh Tung, Ngo Thi Lan, Long Van Duong, Minh Tho Nguyen, Nguyen Minh Tam

**Affiliations:** Faculty of Chemistry, Center for Computational Science, Hanoi National University of Education Hanoi Vietnam; Institute of Materials Science, Graduate University of Science and Technology, Vietnam Academy of Science and Technology 18 Hoang Quoc Viet Hanoi Vietnam; Department of Physics and Technology, Thai Nguyen University of Science Thai Nguyen Vietnam; Institute for Computational Science and Technology (ICST), Quang Trung Software City Ho Chi Minh City Vietnam; Department of Chemistry, KU Leuven Celestijnenlaan 200F B-3001 Leuven Belgium; Computational Chemistry Research Group, Ton Duc Thang University Ho Chi Minh City Vietnam nguyenminhtam@tdtu.edu.vn; Faculty of Applied Sciences, Ton Duc Thang University Ho Chi Minh City Vietnam

## Abstract

Correction for ‘The binary aluminum scandium clusters Al_*x*_Sc_*y*_ with *x* + *y* = 13: when is the icosahedron retained?’ by Ngo Tuan Cuong *et al.*, *RSC Adv.*, 2021, **11**, 40072–40084. DOI: 10.1039/D1RA06994B.

The authors regret that the one of the affiliations (affiliation *b*) was incorrectly shown in the original manuscript. The corrected list of affiliations is as shown above.

The Royal Society of Chemistry apologises for these errors and any consequent inconvenience to authors and readers.

## Supplementary Material

